# The Influence of Systemic Inflammation Response Index on Survival Outcomes of Limited-Stage Small-Cell Lung Cancer Patients Treated with Concurrent Chemoradiotherapy

**DOI:** 10.1155/2020/8832145

**Published:** 2020-12-16

**Authors:** Ahmet Kucuk, Emine Elif Ozkan, Sukran Eskici Oztep, Huseyin Mertsoylu, Berrin Pehlivan, Ugur Selek, Erkan Topkan

**Affiliations:** ^1^Mersin City Education and Research Hospital, Clinic of Radiation Oncology, Mersin, Turkey; ^2^Suleyman Demirel University Medical Faculty, Department of Radiation Oncology, Isparta, Turkey; ^3^Baskent University Medical Faculty, Department of Medical Oncology, Adana, Turkey; ^4^Bahcesehir University, Department of Radiation Oncology, Istanbul, Turkey; ^5^Koc University, School of Medicine, Department of Radiation Oncology, Istanbul, Turkey; ^6^The University of Texas, MD Anderson Cancer Center, Department of Radiation Oncology, Houston, TX, USA; ^7^Baskent University Medical Faculty, Department of Radiation Oncology, Adana, Turkey

## Abstract

**Background:**

Recent studies have indicated that the systemic inflammation response index (SIRI) can efficiently predict survival outcomes in various tumor types. Thusly, in absence of comparable investigations in limited-stage small-cell lung cancers (LS-SCLCs), we aimed to retrospectively evaluate the prognostic utility of SIRI in LS-SCLC patients treated with concurrent chemoradiotherapy (CRT). *Patients and Methods*. Present multi-institutional retrospective analysis incorporated LS-SCLC patients treated with CRT at three academic radiation oncology centers between January 2007 and December 2018. The SIRI was calculated by using the peripheral blood neutrophil (N), monocyte (M), and lymphocyte (L) counts acquired in the last ≤7 days before the commencement of the CRT: SIRI = N × M/L. Accessibility of pretreatment SIRI cutoff that may stratify the study population into two gatherings with distinctive overall survival (OS) results was evaluated by utilizing the receiver operating characteristic (ROC) curve analysis. Primary objective was the association between the SIRI values and the OS results.

**Results:**

Search for the availability of an ideal SIRI cutoff that may stratify the entire patients' population into two particular groups with distinctive OS outcomes identified the 1.93 value (area under the curve (AUC): 72.9%; sensitivity: 74.6%; specificity: 70.1%): Group 1: SIRI <1.93 (*N* = 71) and Group 2: SIRI ≥1.93 (*N* = 110), respectively. At a median follow-up of 17.9 (95% CI: 13.2–22.6) months, 47 (26.0%) patients were still alive (47.9% for SIRI <1.93 versus 18.3% for SIRI ≥1.93; *p* < 0.001). Kaplan–Meier comparisons between the two SIRI groups showed that the SIRI <1.93 cohort had significantly longer median OS (40.5 versus 14.2 months; *p* < 0.001) than the SIRI ≥1.93 cohort. Similarly, the 3- (54% versus 12.6%) and 5-year (33% versus 9.9%) OS rates were also numerically superior in the SIRI <1.93 cohort. Results of the multivariate analyses uncovered that the prognostic significance of the SIRI on OS outcomes was independent of the other confounding variables.

**Conclusions:**

The results of this retrospective multi-institutional cohort analysis suggested that a pre-CRT SIRI was a strong and independent prognostic biomarker that reliably stratified LS-SCLC patients into two cohorts with significantly different OS outcomes.

## 1. Introduction

As a notable cause of cancer-related morbidity and mortality, small-cell lung cancers (SCLCs) represent approximately 15% of all lung cancers [1]. SCLC involves two subgroups per the Veterans Administration Lung Study Group's definition: extensive (ES-SCLC) and limited-stage (LS-SCLC) disease, which accounts for 65% and 35% of all SCLCs, separately [2, 3]. The well-established standard therapy for the LS-SCLC is the cisplatin plus etoposide administered concurrently with thoracic radiotherapy (TRT) followed by prophylactic cranial irradiation (PCI) in patients achieving at least stable disease [4, 5]. Despite attractive initial responses to chemotherapy and TRT, regrettably, the prognoses of such patients are still dismal after aggressive concurrent chemoradiotherapy (CRT), with the respective median and 5-year survival rates of only 16–24 months and 10%–20% [2]. These disappointing outcomes are mainly related to the nondurable response characteristic of the SCLC to the current standard CRT regimens and its notable tendency for fatal widespread distant metastases [2, 6].

Formerly, various clinical indicators have been linked to the prognoses of LS-SCLC patients; including the gender, age, smoking status, performance status (PS), carcinoembryonic antigen (CEA), hemoglobin, alkaline phosphatase (AP), and lactate dehydrogenase (LDH) [2, 7, 8]. However, the SCLC exhibits significantly different response rates and survival times after the standard therapy in patients with practically identical disease stage, prognostic factors, and treatment conditions [9–11]. These differences are chiefly related to the ignorance of the biological markers by the current staging systems, including the recently proposed TNM (tumor-node-metastasis) staging framework [2, 9–12]. Such broad differences assuredly emphasize the compelling demand for the identification of novel biological markers that may serve valuably in better prognostic stratification of the LS-SCLC patients and explicit individualization of their treatment strategies on a per-patient basis.

In this manner, chronic systemic inflammation has been reported to impair the antitumor immune response that promotes tumorigenesis, tumor growth, and metastasis steps [13]. Hence, similar to the other cancers [14–16], the prognostic worth of various bloodborne factors, such as the albumin, C-reactive protein, fibrinogen, and the blood cells either separately or in distinctive blends, to be specific the neutrophil-lymphocyte ratio (NLR), platelet-lymphocyte ratio (PLR), and monocyte-lymphocyte (MLR), GPS, and SII has been extensively examined for their prognostic utility also in LS-SCLC patients [17–26]. The systemic inflammation response index (SIRI), calculated by using the peripheral blood neutrophil, monocyte, and lymphocyte counts, is another easy-to-achieve and cheap biomarker, which has been ascertained to be a solid prognosticator for pancreas-, liver-, nasopharynx-, esophagus-, and renal-cell cancers [27–31]. However, interestingly, the prognostic value of SIRI in LS-SCLC patients has been unquestioned to date. Consequently, in lack of such investigations and essentially based on the credible data proposing an influential prognostic role for SIRI in other cancer sites, we meant to retrospectively assess the prognostic significance of SIRI in LS-SCLC patients treated with concurrent CRT and PCI.

## 2. Patients and Methods

### 2.1. Patient Selection

Between January 2007 and December 2018, we retrospectively evaluated the databases of three independent institutions to identify LS-SCLC patients who underwent CRT followed by PCI for LS-SCLC, namely, the Departments of Radiation Oncology from Baskent University Medical Faculty, Mersin City Education and Research Hospital, and Suleyman Demirel University Medical Faculty. Inclusion criteria were as follows: aged 18 to 80 years, Eastern Cooperative Oncology Group (ECOG) performance score of 0–2, histopathological proof of proven SCLC, staged as LS-SCLC according to VALSG criteria using thoracic computerized tomography (CT) and 18F-fluorodeoxyglucose positron emission tomography-CT (PET-CT), available pre-CRT brain magnetic resonance imaging scans (MRI) at the past 30 days, detailed CRT records and computerized treatment data sets of TRT, available complete blood count and biochemistry test results obtained within the pre-CRT 7-days period, available complete follow-up, and survival data. Patients with a history of infectious disease and/or severe infections at past 30 days before the onset of CRT, previous history of immunologic disorders, chronic or continuous use of immune suppressants or steroids, presence of malignant pleural/pericardial effusions, inadequate pulmonary, cardiac, renal, and/or hepatic functions, previous history of RT/chemotherapy, and those patients with missing CRT, PCI, or follow-up/survival data were excluded from this analysis.

The study design was approved by the institutional review board of Baskent University Medical Faculty before the acquisition of any patient data, and written informed consent was provided by each participant either themselves or legally authorized representatives for collection and analysis of blood samples, pathologic specimens, and academic publication of their outcomes.

### 2.2. Concurrent Chemoradiotherapy

All patients underwent PET-CT fusion-based RT planning as intimated by all three institutions' LS-SCLC management standards. For TRT, all cases received 3-dimensional conformal RT (3D-CRT) or intensity-modulated RT (IMRT) using megavoltage linear accelerators. Patients received 4 courses of chemotherapy (cisplatin 60 mg/m^2^ i.v. on day 1 and etoposide 120 mg/m^2^ i.v. on days 1, 2, and 3 given every 3 weeks) and underwent concurrent TRT either beginning with the 1st or 2nd chemotherapy courses. TRT was applied using one of the total doses of 45 Gy bid (1.5 Gy/fx, twice daily, 30 fractions in 15 days) or conventionally fractionated 54 Gy (1.8 Gy/fx, 30 days).

### 2.3. Measurement of SIRI

The SIRI values were calculated per Qi's original formula [27]: SIRI =N × M/L, by utilizing each patient's neutrophil, monocyte, and lymphocyte measures acquired in the past ≤7 days before the commencement of the CRT.

### 2.4. Response Assessment, PCI, and Follow-Up

All patients were first re-evaluated with chest and abdomen CT scans, and brain MRI to determine the treatment response at the 6th week of completion of CRT. The patients who were considered to achieve at least stable disease per PERCIST criteria with no apparent clinical or radiological evidence of brain metastases and no proof of severe neurological disorders underwent a PCI protocol consisting of a total dose of 25 Gy (2.5 Gy/fx, 10 days), if not rejected. Furtherly, patients were evaluated for response at 3 monthly (first 2 years), 6 monthly (3rd to 5th years), and yearly interims thenceforth. Patient evaluations incorporated the blood count/chemistry and PET-CT or chest CT scans (after confirmed complete response on PET-CT). Additional restaging tools were utilized only if indicated.

### 2.5. Statistical Analysis

The primary object of the study was to assess the association between the SIRI and OS (defined as the interval between the first day of CRT and the date of death/last visit) results. Quantitative variables were described by medians and ranges, while frequencies and percentages were used to outline the categorical variables. Chi-square test, Student's *t*-test, Fisher's exact test, or Spearman's correlation estimates were used to compare frequency distributions, as indicated. Accessibility of pretreatment SIRI cutoff(s) that may stratify the study population into two gatherings with distinctive treatment response was evaluated by using the receiver operating characteristic (ROC) curve analysis. The OS times were estimated with using the Kaplan–Meier method, and the significance of differences between the groups was measured with the log-rank test. The Cox proportional-hazards model was adopted for the multivariate analysis by including only the variables exhibiting significance in univariate analysis. All *P* values were 2-tailed and were considered significant if <0.05.

## 3. Results

A sum of 181 consequently treated LS-SCLC patients from three radiation oncology centers were included in this retrospective cohort analysis. As depicted in Table 1, the median age was 60 years (range: 33–79) with males (85.0%) and ex-smokers (96.1%) accounting for the considerable majority of the study population. Ay baseline, 60.2% of patients were anemic according to the standard World Health Organization definition. The TRT protocol was HFRT of 45 Gy and 3D-CRT of 54 Gy in 57.4% and 42.6% patients, respectively. PCI was received by 139 (76.7%) patients, while respective 23 (12.7%) and 19 (10.6%) patients did not receive PCI due to self-refusal or presence of chronic vascular or neurological disorders.

**Figure 1:**
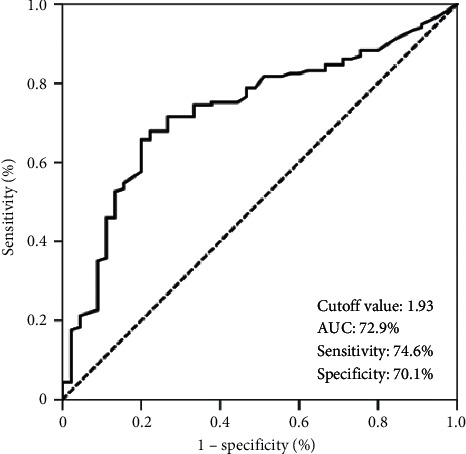
Results of receiver operating characteristic curve analyses: overall survival.

Search for the availability of an ideal SIRI cutoff that may stratify the entire cohort into two particular groups with significantly distinctive OS outcomes identified the 1.93 value (area under the curve (AUC): 72.9%; sensitivity: 74.6%; specificity: 70.1%): Group 1: SIRI <1.93 (*N* = 71) and Group 2: SIRI ≥1.93 (*N* = 110), respectively (Figure 1). Direct comparisons between the two SIRI groups per baseline patients' demographics and treatment features revealed no notable discrepancies among them (Table 1).

**Figure 2:**
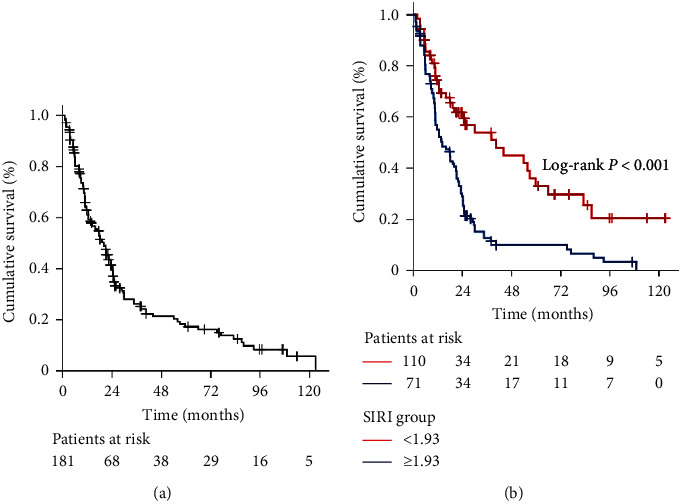
Overall survival outcomes: (a) whole-study population and (b) per pretreatment SIRI groups (blue line: SIRI < 1.9 and green line: SIRI ≥  1.9).

At a median 17.9 (95% CI: 13.2–22.6) months of follow-up, 47 (26.0%) patients were still alive (47.9% for SIRI <1.93 versus 18.3% for SIRI ≥1.93; *p* < 0.001). The median, 3- and 5-year OS rates were 20.3 months (95% CI: 15.3–25.3 months), 25.9% and 17.1% for the entire research cohort (Figure 2(a)). Kaplan–Meier comparisons between the two SIRI cohorts revealed that the SIRI <1.93 cohort had significantly longer median OS (40.5 versus 14.2 months; *p* < 0.001) than its SIRI ≥1.93 counterpart. Similarly, the 3- (54% versus 12.6%) and 5-year (33% versus 9.9%) OS rates were also numerically superior in the SIRI <1.93 cohort (Figure 2(b)).

In univariate analysis, the ECOG performance status 0–1 (versus 2; *p*=0.004), absence of anemia (versus presence; *p*=0.028), to receive PCI (versus no PCI; *p*=0.003), to receive TRT with HFRT schedule (versus CFRT; *p*=0.003), and SIRI <1.93 (versus SIRI ≥1.93; *p* < 0.001) emerged to be the factors to connect with significantly superior OS times. Results of the multivariate analyses uncovered that all factors retained their independent significance (Table 2).

## 4. Discussion

The present retrospective study examined the prognostic quality of SIRI in a group of 181 LS-SCLC patients from three radiation oncology centers. To our best information, this is the first multi-institutional study to objectively analyze the probable relationship between the SIRI and OS outcomes in patients with LS-SCLC undergoing CRT and PCI. Other than asserting the profound significance of HFRT and PCI, our results exhibited a meaningful link between the pre-CRT SIRI ≥1.93 and diminished OS outcomes (*p* < 0.001) in these patients' groups.

Albeit each of neutrophil, monocyte, and lymphocyte counts demonstrate critical prognostic significance in various cancer types, yet the novel SIRI is a more potent prognosticator than either of the cell types alone or their two-cell combinations [27–31]. This is principally due to SIRI's capacity to fully assess the balance between host immune and inflammatory conditions by consolidating all three cells simultaneously in the formula. Besides, the integrated SIRI is less likely to be influenced by various conditions; acute or chronic infections, liver and bone diseases, fluid retention, and dehydration contrasted with its individual cell components. Hence, it is prudent to envision that SIRI has a more robust and reliable prognostic utility in cancer patients, including the LS-SCLC patients, which framed a reasonable basis for conduction of this present investigation in the absence of similarly designed studies.

Our present results confirmed the prognostic value of better performance score [32], absence of anemia [33, 34], use of HFRT scheme [4, 35], and PCI [5] in the successful management of the LS-SCLCs. However, the most remarkable finding of the current investigation was the exhibit of the SIRI ≥ 1.93 as an innovative surrogate marker of significantly shorter median OS (14.2 versus 40.5 months; HR: 3.09; *p* < 0.001) times as opposed to the SIRI < 1.93. Also, attesting the long-term endurance of the negative impact of severe systemic inflammatory conditions, the SIRI ≥ 1.93 cohorts demonstrated a notably lesser chance of surviving beyond 5 years (9.9% versus 36.1% for SIRI < 1.93). Although it is strenuous to competently discuss present results comparatively in the lack of similar SIRI studies, yet they have all the earmarks of being in good accordance with the results of previous SIRI reports for other tumor sites [27–31] as well as the systemic inflammation or immune-inflammation marker studies in SCLC patients [17–26]. Qi et al. [27] represent the first to introduce the potential link between the SIRI and cancer prognosis in 2016 by disclosing the connection between a high SIRI level and elevated serum inflammatory cytokine/chemokine measures, and resultant reduced progression-free survival and OS times in pancreatic cancer patients. Given these results, the researchers proposed that the predictive ability of a high SIRI value was more robust than other accessible inflammation indexes regarding the chemotherapy resistance due to the presence of an exacerbated chronic inflammatory status and/or massive disease burden. Positively confirming this proposal, several consecutive researchers demonstrated statistically meaningful correlations between the high SIRI levels and pathological tumor grade, tumor size, overall TNM stage, lymphatic and vascular invasion status, progesterone receptor status in various cancers [28, 36, 37], and worsened locoregional/distant relapse and survival results [28, 36–38].

Since the SIRI has never been investigated in LS-SCLC patients before, it might be rational to compare our results with the other immune and inflammation markers reported for SCLC patients. One such firm marker is the SII, where just the platelets replace monocytes of the SIRI (SII = platelets × neutrophils/lymphocytes). In a 2015 study, Hong et al. [39] analyzed the outcomes of 919 SCLC patients to reveal the link between the various pretreatment inflammation-based scores and the clinical outcomes. The results of this study demonstrated the superiority of SII over the NLR, PLR, and PNI in prognostic stratification of LS-SCLC patients (*N* = 552), which might be associated with the SII's superior ability to reflect the patients' immune and inflammatory status simultaneously. Latterly, Yang et al. [40] were able to demonstrate a significant connection between high SII values and essentially diminished progression-free survival (*p* < 0.001), and OS (*p* < 0.001) results in a sum of 228 SCLC patients. Likewise, Wang et al. [41] intended to assess the prognostic usefulness of the pretreatment SII in SCLC patients treated with the standard etoposide and platinum-based chemotherapy (*N* = 653). The median OS was significantly longer in the low-SII group than the high-SII group (17.0 versus 12.0 months; *p* < 0.001) in this study. These results were further confirmed by the results of two comprehensive meta-analyses comprising SCLC and other tumor primaries [40, 42]. Because both the platelets of the SII and the monocytes of the SIRI immunologically behave as tumor-promoting factors in specific ways, the remarkable concordance between the previously reported SII studies and our novel SIRI research appears to suggest both biological indices as potent indicators of LS-SCLC prognosis.

Our study is strengthened by representing the outcomes of a multi-institutional cohort study exclusively consolidating the patients who underwent standard pretreatment FDG-PET-CT and cranial MRI staging procedures. Yet, the present study also has several impediments: first, it was a retrospective cohort analysis in a relatively small LS-SCLC population size, where unpredictable biases may have adjusted the ultimate outcomes in favor of either SIRI group. Accordingly, present findings should be interpreted with great caution until its results become confirmed with prospectively planned large-scale investigations. Second, our study population included heterogeneously treated patients with regards to the TRT technique, total dose, dose per fraction, and fractionation, as well as the use of PCI. On the other hand, this may likewise be a remarkable advantage by reflecting the real-world experience as opposed to the observations in a homogeneously treated patients' population. These apparent discrepancies may also propose an independent significance for SIRI in prognostic stratification of LS-SCLC patients irrespective of the treatment protocols and, therefore, its broader applicability. Third, as a biasing factor, our present conventionally fractionated 54 Gy dose may be noticed as minimal compared to the CONVERT (concurrent once-daily versus twice-daily radiotherapy) trial's 66 Gy [35]. However, the SIRI's prognostic robustness was independent of the TRT scheme, as revealed from the multivariate analysis outcomes. These results collectively suggest the patients' actual biological condition as an essential determinant of the outcomes irrespective of the treatment regime, at least up to 66 Gy of TRT. And fourth, we chose a single time-point cutoff despite the SIRI's highly dynamic nature that may demonstrate notable fluctuations during the CRT or post-treatment follow-up periods leading to potential alterations in the calculated SIRI cutoffs. For a vital example, both the RT and chemotherapy remain well-established treatments inducing significant reductions in the blood cells, such as the relative or absolute lymphopenia [43, 44]. Accordingly, future research focusing on the SIRI dynamics may contribute valuable information about the fittest time interval for defining the most suitable SIRI cutoff, like the nadir SIRI. Despite these overcome obstacles, high SIRI values appeared to indicate an immunologically suppressed and severely aggravated inflammatory condition. Hence, our current results seem to recommend the pretreatment SIRI as a stable biomarker, which may serve usefully in the judicious selection of the best-fit treatments on a per-patient basis by stratifying the LS-SCLC patients into prognostically separate groups.

## 5. Conclusions

The results of this retrospective multi-institutional cohort analysis suggested the pre-CRT SIRI ≥1.93 score as a firm and independent prognostic biomarker that dependably stratified the LS-SCLC patients into two cohorts with distinct OS outcomes. If asserted with future research results, high SIRI values might serve usefully in the reliable identification of patients with dismal prognoses and their individualized treatments in routine clinical practice, since the novel SIRI is a widely accessible, almost costless, objectively measurable, and replicable biological marker.

## Figures and Tables

**Table 1:** Baseline demographics and treatment characteristics for the entire study population and per SIRI groups.

Characteristic	All patients (*N* = 181)	SIRI ≥ 1.93 (*N* = 110)	SIRI < 1.93 (*N* = 71)	*p* value
Median age, y (range)	60 (33–80)	61 (33–79)	59 (36–79)	0.91

*Age group, y (%)*				
<70 years	132 (72.9)	81 (73.6)	51 (71.8)	0.87
≥70 years	49 (27.1)	29 (26.4)	20 (28.2)	

*Gender, n (%)*				
Male	154 (85.0)	91 (82.7)	63 (88.7)	0.39
Female	27 (15.0)	19 (17.3)	8 (11.3)	

*ECOG-PS, n (%)*				
0–1	141 (71.7)	84 (76.4)	57 (80.3)	0.48
2	40 (28.3)	26 (23.6)	14 (19.7)	

*Smoking status, n (%)*				
Ex-smoker	174 (96.1)	106 (96.3)	68 (95.8)	0.90
Current smoker	7 (3.9)	4 (3.7)	3 (4.2)	

^*∗*^ *Anemia, n (%)*				
Present	109 (60.2)	66 (60.0)	43 (60.6)	0.94
Absent	72 (39.8)	44 (40.0)	28 (39.4)	

*PCI, n (%)*				
Yes	139 (76.7)	85 (77.3)	54 (76.1)	0.79
No	42 (23.3)	25 (22.7)	17 (23.9)	

*TRT scheme*				
CFRT 54 Gy	77 (42.6)	47 (42.7)	30 (42.3)	0.86
HFRT 45 Gy	104 (57.4)	63 (57.3)	41 (57.7)	

*Treatment center*				
Baskent Un.	104 (57.4)	63 (57.3)	41 (57.7)	0.74
Mersin City Hospital	56 (30.9)	35 (31.8)	21 (29.6)	
Suleyman Demirel Un.	21 (11.7)	12 (10.9)	9 (12.7)	

^*∗*^Anemia refers to any Hb <130 g/dL for males and Hb <120 g/dL for females. *Abbreviations *. SIRI: systemic immune-inflammation response index; ECOG-PS, Eastern Cooperative Oncology Group performance status; PCI: prophylactic cranial irradiation; CFRT: conventionally fractionated radiotherapy; HFRT: hyperfractionated radiotherapy; Gy: gray; Un.: university.

**Table 2:** Results of univariate and multivariate analyses.

Characteristic	Patients (*N*)	Median OS (months)	Univariate *p* value	HR	Multivariate *p* value
*Age group*					
<70 years	132	23.3	0.18	1.08	0.29
≥70 years	49	17.7			

*Gender*					
Male	154	18.1	0.11	1.06	0.21
Female	27	23.8			

*Smoking status*					
Ex-smoker	174	20.5	0.54	1.05	0.69
Current smokers	7	19.5			

*ECOG-PS*					
0–1	141	22.4	0.004	1.76	0.026
2	40	15.8			

*Anemia*					
Absent	72	23.7	0.028	1.37	0.041
Present	109	17.6			

*PCI*					
Yes	139	20.8	0.003	1.93	0.027
No	42	11.8			

*TRT scheme*					
HFRT 45 Gy	104 (57.4)	23.4	0.003	1.59	0.017
CFRT 54 Gy	77 (42.6)	15.8			

*SIRI*					
<1.93	110	40.5	<0.001	3.02	<0.001
≥1.93	71	14.2			

*Abbreviations*. OS: overall survival; HR: hazard ratio; ECOG-PS, Eastern Cooperative Oncology Group performance status; PCI: prophylactic cranial irradiation; HFRT: hyperfractionated radiotherapy; CFRT: conventionally fractionated radiotherapy; SIRI: systemic immune-inflammation response index.

## Data Availability

The datasets used and/or analyzed during the current study are available from the Baskent University Department of Radiation Oncology Institutional Data Access for researchers who meet the criteria for access to confidential data, contact address: adanabaskent@baskent.edu.tr.
